# Omega-3 and omega-6 DPA equally inhibit the sphingosylphosphorylcholine-induced Ca^2+^-sensitization of vascular smooth muscle contraction via inhibiting Rho-kinase activation and translocation

**DOI:** 10.1038/srep36368

**Published:** 2017-02-07

**Authors:** Ying Zhang, Min Zhang, Bochao Lyu, Hiroko Kishi, Sei Kobayashi

**Affiliations:** 1 Department of Molecular and Cellular Physiology, Yamaguchi University, Graduate School of Medicine, 1-1-1 Minami-Kogushi, Ube, Yamaguchi 755-8505, Japan

## Abstract

We previously reported that eicosapentaenoic acid (EPA), an omega-3 polyunsaturated fatty acid (n-3 PUFA), effectively inhibits sphingosylphosphorylcholine (SPC)-induced Ca^2+^-sensitization of vascular smooth muscle (VSM) contraction which is a major cause of cardiovascular and cerebrovascular vasospasm, and EPA is utilized clinically to prevent cerebrovascular vasospasm. In this study, we clearly demonstrate that docosapentaenoic acid (DPA), which exists in two forms as omega-3 (n-3) and omega-6 (n-6) PUFA, strongly inhibits SPC-induced contraction in VSM tissue and human coronary artery smooth muscle cells (CASMCs), with little effect on Ca^2+^-dependent contraction. Furthermore, n-3 and n-6 DPA inhibited the activation and translocation of Rho-kinase from cytosol to cell membrane. Additionally, SPC-induced phosphorylation of myosin light chain (MLC) was inhibited in n-3 and n-6 DPA pretreated smooth muscleVSM cells and tissues. In summary, we provide direct evidence that n-3 and n-6 DPA effectively equally inhibits SPC-induced contraction by inhibiting Rho-kinase activation and translocation to the cell membrane.

Vasospasm of vascular smooth muscle (VSM), including cardiovascular and cerebrovascular vasospasm, remains a significant source of morbidity and mortality in sudden death and in patients after subarachnoid hemorrhage (SAH). Although the exact mechanism by which VSM produces a vasospasm remains unclear, studies have suggested that Rho-kinase mediated Ca^2+^-sensitization of VSM contraction is associated with coronary artery and cerebral vasospasm^1^,^2^,^3^,^4^,^5^. We previously demonstrated^6^ that sphingosylphosphorylcholine (SPC) induced Ca^2+^-sensitization of VSM contraction in arterial strips by activating Rho-kinase^7^,^8^,^9^. Subsequently, eicosapentaenoic acid (EPA), one of omega-3 polyunsaturated fatty acids (n-3 PUFAs) from fish, was found to inhibit the SPC-induced Ca^2+^-sensitization of VSM contraction with no effect on Ca^2+^-dependent normal contraction, which plays an important role in the maintenance of physiologic blood pressure^10^.

The two families of PUFAs, omega-3 (n-3) and omega-6 (n-6) PUFAs, are classified based on the location of the last double bond relative to the terminal methyl group of the molecule^11^,^12^. Accumulating evidence demonstrates that n-3 PUFAs, including EPA and docosahexaenoic acid (DHA) from fish and fish oils, are beneficial in prevention of cardiovascular diseases^13^,^14^,^15^,^16^,^17^. Conversely, n-6 PUFAs, such as arachidonic acid (AA; 20:4 n-6), which is the substrate for the synthesis of a variety of proinflammatory and vasoconstrictive molecules, are believed to be proinflammatory^18^,^19^,^20^. Some studies indicate that n-6 PUFAs have anti-inflammatory properties^21^,^22^,^23^,^24^, and higher consumption of n-6 PUFAs appears safe and may reduce the risk of cardiovascular disease^25^,^26^.

We have demonstrated that n-3 PUFAs, such as EPA and DHA, inhibited SPC-induced Ca^2+^-sensitization of VSM contraction^27^, however, it is unknown whether DPA, an intermediate product between EPA and DHA, is capable of inhibiting SPC-induced VSM contraction. Unlike EPA and DHA, DPA has two forms, n-3 DPA and n-6 DPA^28^,^29^. The n-3 and n-6 DPA are reported to be beneficial in improving the lipoprotein profile and aortic function in hamsters fed a high cholesterol diet^30^. Rissanen *et al*. also reported that a high proportion of fish-derived DHA and DPA in serum is associated with a decreased risk of acute coronary syndrome^31^.

Although some studies have evaluated the link between n-3 DPA and the risk for cardiovascular disease, there is no direct evidence on whether n-3 and n-6 DPA affect Ca^2+^-sensitization of VSM contraction. Specifically, no study has investigated the effect of n-6 PUFAs on VSM contraction. The purpose of the present study was to (i) investigate and compare the effects of individual n-3 DPA and n-6 DPA on SPC-induced Ca^2+^-sensitization and Ca^2+^-dependent contraction of VSM; (ii) determine the effects of n-3 and n-6 DPA on intracellular [Ca^2+^] by simultaneously measuring [Ca^2+^]i and VSM contraction; and (iii) examine the mechanism of how n-3 and n-6 DPA affect SPC-induced Ca^2+^-sensitization of VSM contraction.

## Results

### Effects of n-3 and n-6 DPA on SPC and 40 mM K^+^ depolarization-induced contraction of VSM

**Figure 1:**
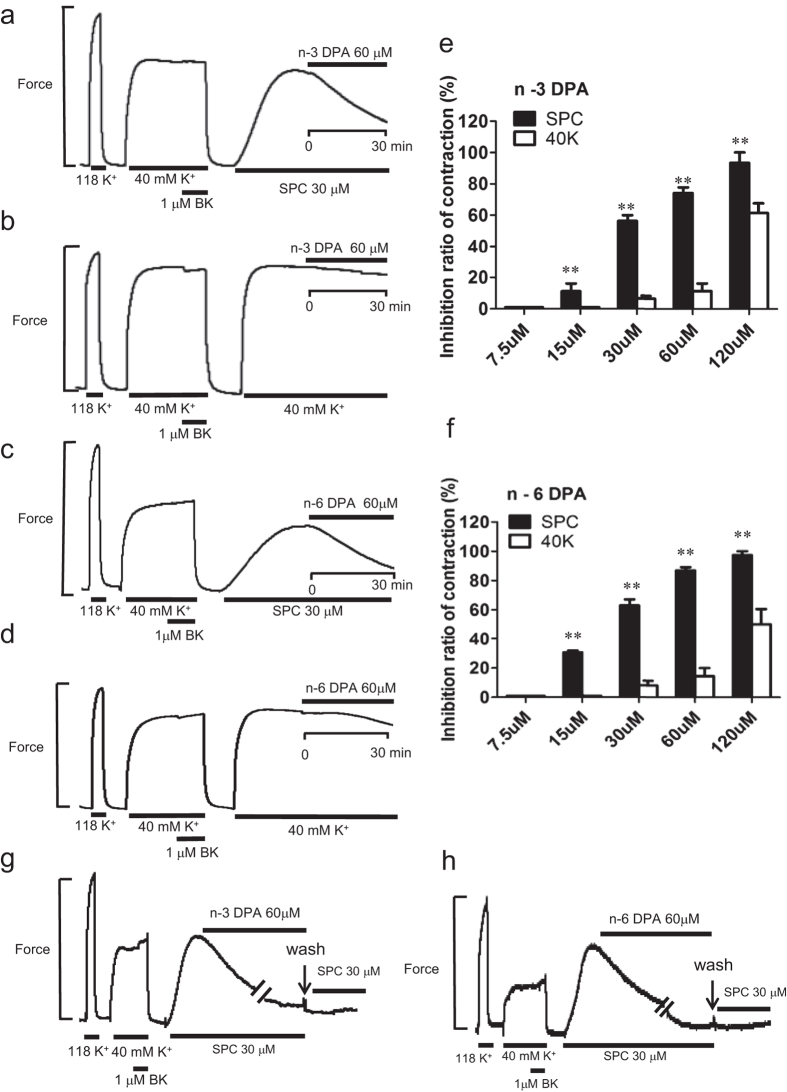
Effects of n-3 and n-6 DPA on SPC and 40 mM K^+^ depolarization-induced contraction of VSM. (**a,c**) Representative recordings showing the direct effects of 60 μM n-3 and n-6 DPA on SPC-induced contraction of vascular smooth muscle (VSM), respectively. (**b**) and (**d**) Representative recordings showing the direct effects of 60 μM n-3 and n-6 DPA on 40 mM K^+^ depolarization-induced contraction of VSM, respectively. (**e**,**f**) Dose dependence of inhibitory ratio of n-3 and n-6 DPA on SPC and 40 mM K^+^ depolarization-induced contraction of VSM (n ≧ 4, **P < 0.01). (**g**,**h**) Representative recordings showing the irreversible effects of n-3 and n-6 DPA on SPC-induced contraction of VSM, respectively.

**Figure 2:**
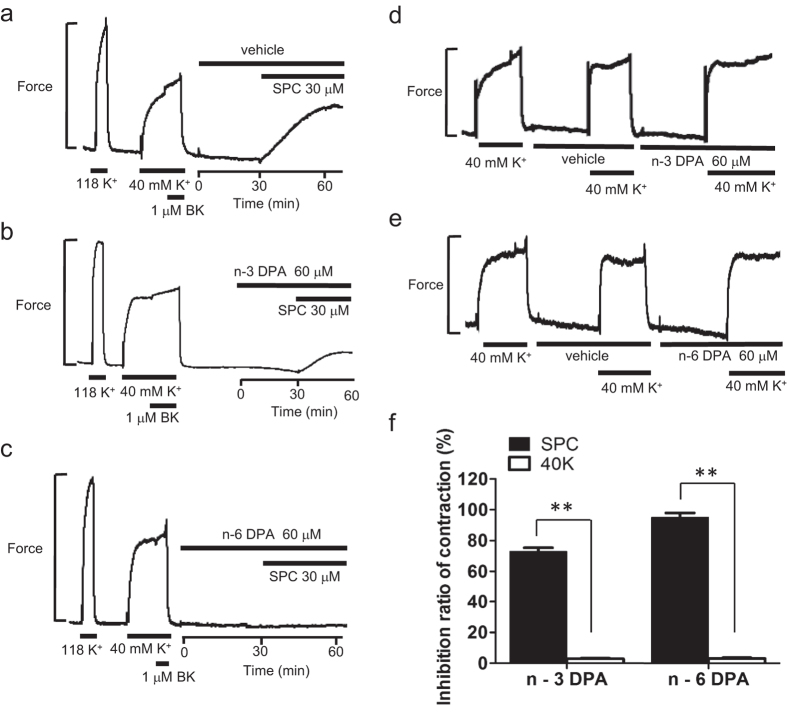
Effects of n-3 and n-6 DPA pretreatment on SPC and 40 mM K^+^ depolarization-induced contraction of VSM. Representative recordings showing the effects on SPC -induced contraction of VSM in vehicle control (**a**) n-3 DPA pretreatment (**b**) and n-6 DPA pretreatment (**c**). Representative recordings showing the effects on 40 mM K^+^ depolarization-induced contraction of VSM in n-3 DPA pretreatment (**d**) and n-6 DPA pretreatment (**e**) after vehicle pretreatment. (**f**) Effects of pretreatment with n-3 DPA and n-6 DPA on SPC and 40 mM K^+^ depolarization-induced contraction of VSM (n = 3, **P < 0.01).

The chemical structures of n-3 and n-6 DPA are shown in Supplementary Fig. S1. First, we observed the direct effects of DPA on SPC-induced contraction in tissue strips. Both n-3 and n-6 DPA (60 μM) strongly inhibited SPC-induced Ca^2+^-sensitization of VSM contraction by 74.03 ± 6.42% and 86.87 ± 3.74%, respectively, (Fig. 1a,c), compared with vehicle control (see Supplementary Fig. S2). The inhibitory effects of n-3 and n-6 DPA on SPC-induced contraction were dependent on concentration (Fig. 1e,f). In contrast, n-3 and n-6 DPA (60 μM) showed little inhibitory effect (11.48 ± 8.41% and 14.57 ± 9.71%, respectively) in 40 mM K^+^ depolarization-induced contraction of VSM (Fig. 1b,d) compared with vehicle control (see Supplementary Fig. S2). Next, we investigated the effects of DPA pretreatment on SPC-induced contraction in tissue strips. After pre-treating the tissue strips with DPA for 30 min, SPC was added to the media to stimulate contraction. SPC stimulation caused a minor contraction in tissue strips pretreated with 60 μM n-3 and n-6 DPA compared with strong contraction in vehicle control (Fig. 2a–c), showing that pretreatment with n-3 and n-6 DPA significantly inhibited SPC-induced contraction by 73.00 ± 2.31% and 95.00 ± 2.89%, respectively (Fig. 2f). Pretreatment with DPA did not affect the 40 mM K^+^ depolarization-induced contraction of VSM (Fig. 2d,e). We also observed that the inhibitory effect of DPA is irreversible when we applied SPC (without DPA) to the media after we observed a direct inhibitory effect on SPC-induced contraction by DPA after washout for 30 minutes (Fig. 1g,h).

### Effects of n-3 and n-6 DPA on [Ca^2+^]_i_ level during SPC and 40 mM K^+^ depolarization-induced contraction of VSM

**Figure 3:**
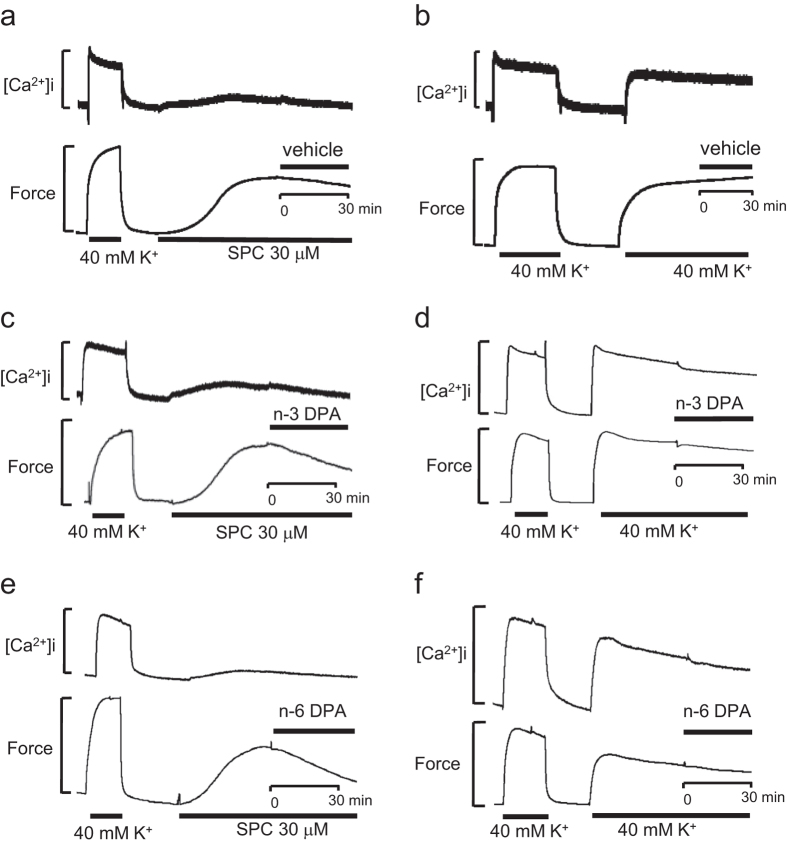
Simultaneous measurements of [Ca^2+^]i and force in fura-2-loaded coronary arterial strips after stimulation with SPC and 40 mM K^+^. Representative recordings showing the effects on [Ca^2+^]i and force after stimulation with SPC in the presence of vehicle (**a**) n-3 DPA (**c**) and n-6 DPA (**e**). Representative recordings showing the effects on [Ca^2+^]i and force after stimulation with 40 mM K^+^ in the presence of vehicle (**b**) n-3 DPA (**d**) and n-6 DPA (**f**).

Because n-3 and n-6 DPA slightly inhibited the 40 mM K^+^ depolarization-induced contraction of VSM, we wished to assess whether they could affect the levels of [Ca^2+^]i. To investigate whether n-3 and n-6 DPA alter the levels of [Ca^2+^]i, we simultaneously assessed changes in force and [Ca^2+^]i. As shown in Fig. 3c,e, SPC induced the contraction of VSM with little or no change in the concentration of Ca^2+^ compared with vehicle control (Fig. 3a), which was consistent with a previous report^6^,^8^. Additionally, n-3 and n-6 DPA inhibited the SPC-induced contraction of VSM without changing the concentration of Ca^2+^ compared with that of vehicle control-treated VSM (Fig. 3a), suggesting that these inhibitory effects on SPC-induced contraction of VSM were Ca^2+^-independent. Conversely, n-3 and n-6 DPA slightly inhibited the 40 mM K^+^ depolarization-induced contraction of VSM and the level of [Ca^2+^]i (Fig. 3d,f) compared with vehicle control-treated VSM (Fig. 3b). Based on these results, the slight inhibition of the [Ca^2+^]i level by n-3 or n-6 DPA could account for their inhibition of the 40 mM K^+^ depolarization-induced contraction of VSM. However, the inhibitory effects of n-3 and n-6 DPA on SPC-induced contraction of VSM are Ca^2+^-independent.

### Effects of direct pretreatment with n-3 and n-6 DPA on VSM cell contraction induced by SPC

**Figure 4:**
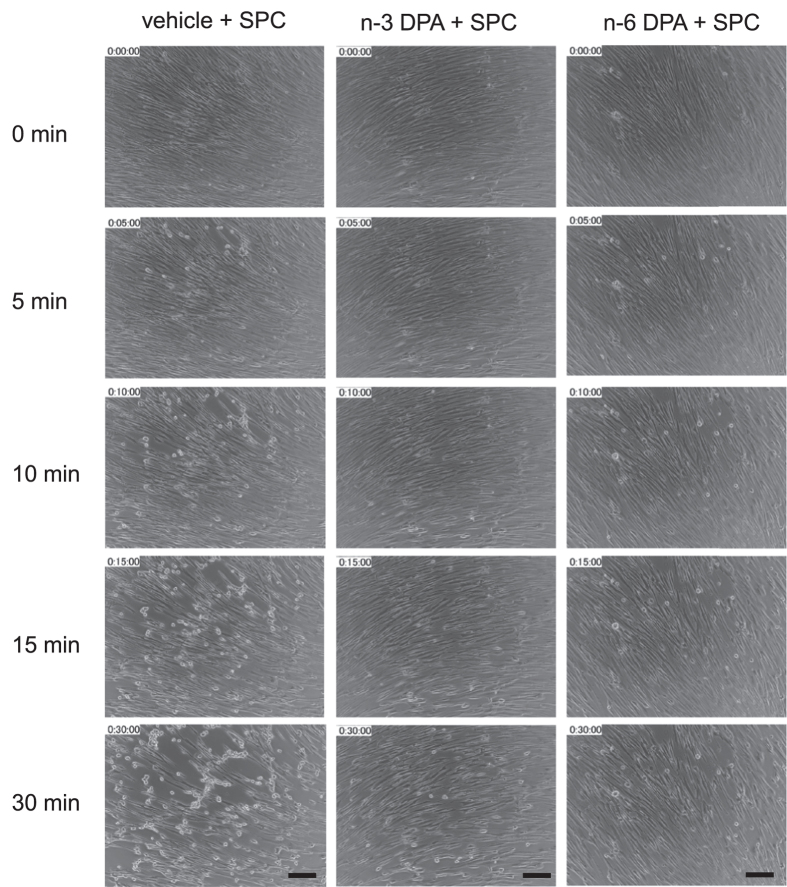
Live cell imaging of cellular contraction upon stimulation with SPC in CASMCs in the absence or presence of n-3 or n-6 DPA. Representative images of cells pretreated with n-3 or n-6 DPA and vehicle control after stimulation with SPC for different lengths of time. Scale bar = 10 μm.

To further investigate the mechanism of n-3 and n-6 DPA on SPC-induced VSM contraction, we used cultured coronary artery smooth muscle cells (CASMCs). First, we examined the effects of direct pretreatment with n-3 and n-6 DPA on SPC-induced contraction of CASMCs by recording the time course of changes in CASMC cell morphology. Based on previous reports^32^,^33^, after the cells were 80–90% confluent, the cultures were switched to serum-free and growth factor-free media. After 2 days, a spindle-shaped, contractile type of cells was observed (see Supplementary Fig. S3). Time-lapse observations recorded that a contraction in CASMCs was induced by SPC in the absence and presence of n-3 or n-6 DPA. As shown in Fig. 4, SPC induced a noticeable contraction in CASMCs, with the elongated cells becoming short or round (see Supplementary Video 1). In contrast, CASMCs pretreated with n-3 or n-6 DPA showed little change in morphology after being stimulated with SPC for 30 min (see Supplementary Videos 2 and 3).

**Figure 5:**
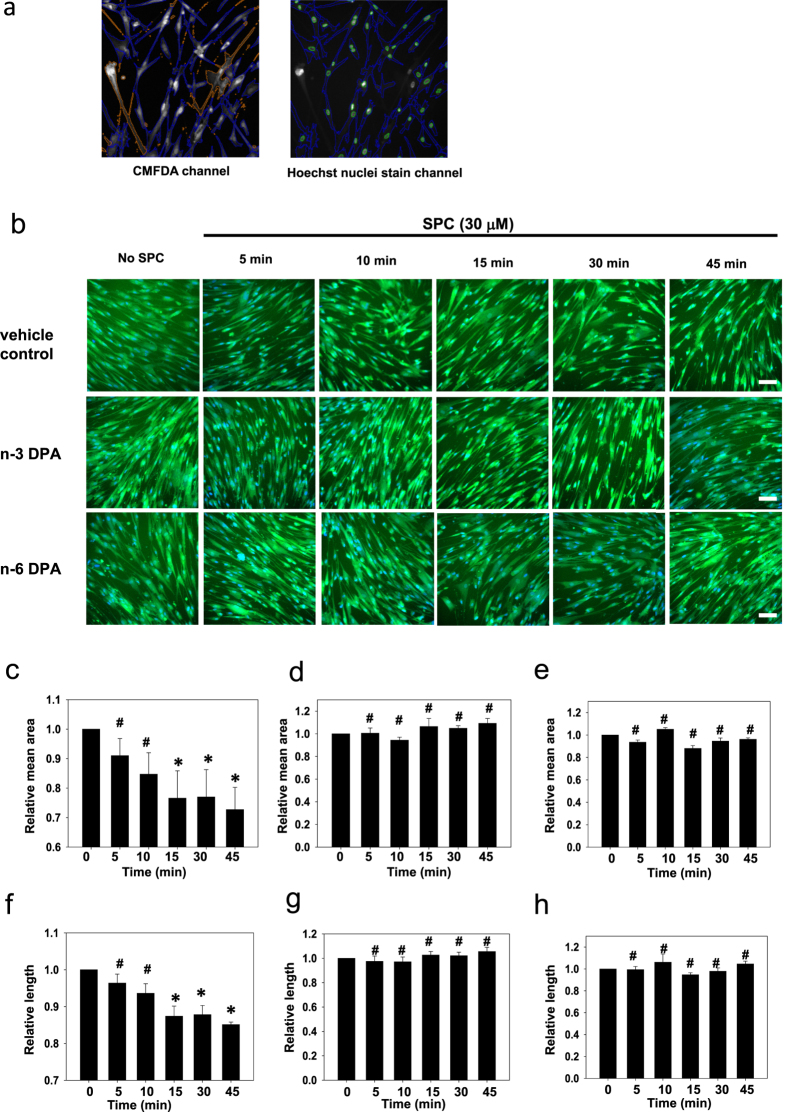
Effects of n-3 and n-6 DPA on SPC-induced contraction in CASMCs visualized by ArrayScan. (**a**) Definition of the area and number of cells with ArrayScan™ Software. The blue lines represent an individual cell based on CMFDA cytoplasmic staining (left and right images), and green lines represent cell nuclei based on Hoechst nuclear staining (right image) according to the settings of the Bioapplication (Thermo Fisher Scientific) algorithm. (**b**) Representative images of wells with vehicle control pretreatment, n-3 DPA pretreatment (60 μM for 30 min), and n-6 DPA pretreatment (60 μM for 30 min) after stimulation with SPC for different lengths of time. Scale bar = 20 μm. Statistical analysis of SPC-stimulation-induced changes in the cell area of the vehicle control group (**c**) n-3 DPA pretreated group (**d**) and n-6 DPA pretreated group (**e**) after different times of SPC stimulation. Statistical analysis of SPC-stimulation-induced changes in the cell length of the vehicle control group (**f**) n-3 DPA pretreated group (**g**) and n-6 DPA pretreated group (**h**) after different times of SPC stimulation. Data are expressed as the mean±SE. *P < 0.01 versus 0 min, ^#^*P* > 0.05 versus 0 min.

We then used the ArrayScan V^TI^ High Content Screening Reader to analyze and quantify changes in cellular area and length induced by SPC-stimulation in CASMCs pretreated with DPA or vehicle control. As shown in Fig. 5, after stimulation with SPC, vehicle control-treated CASMCs began to contract. The area and length of CASMCs decreased to 72.7 ± 7.56% and 85.1 ± 0.69% compared with those of no SPC-stimulated cells (100%) during the 45 min stimulation with SPC (Fig. 5b,c,f). However, the area and length of cells pretreated with n-3 or n-6 DPA did not change during the 45 min stimulation with SPC, indicating that n-3 or n-6 DPA inhibited SPC-induced contraction in CASMCs (Fig. 5b,d,e,g,h).

### n-3 and n-6 DPA inhibited Rho-kinase translocation induced by SPC in VSM cells

**Figure 6:**
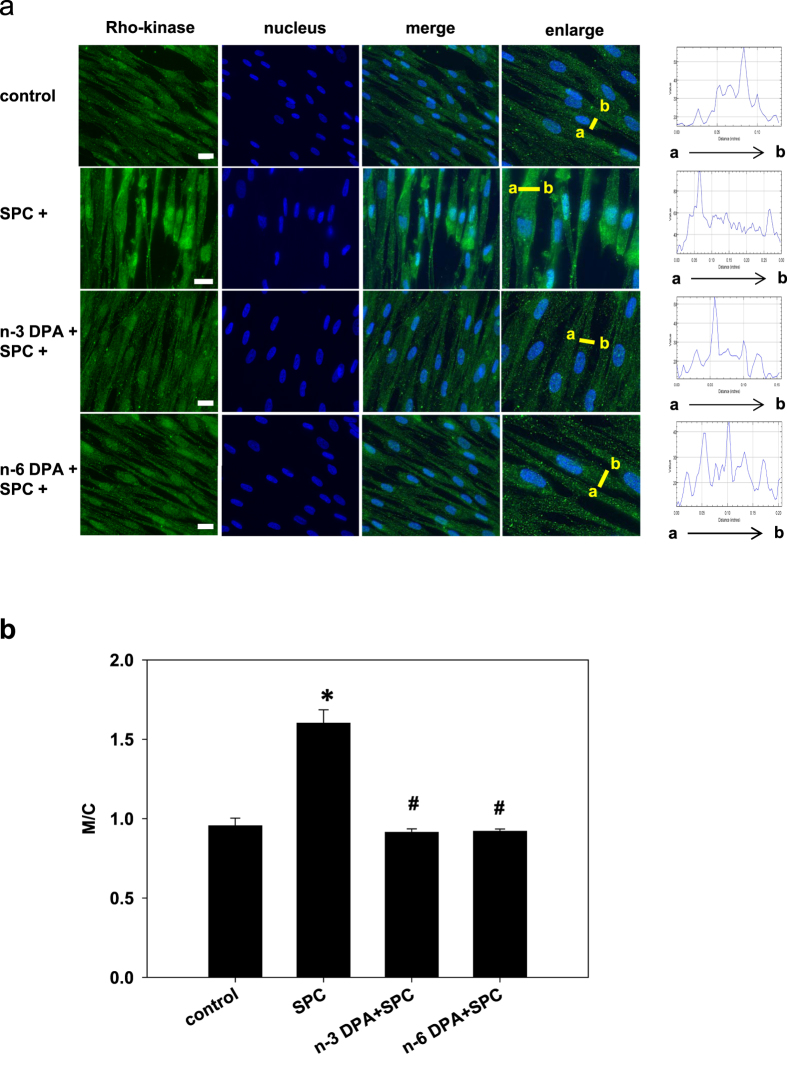
The inhibitory effects of n-3 and n-6 DPA on SPC-induced Rho-kinase translocation. (**a**) Representative images of cells pretreated with n-3 or n-6 DPA (60 μM) or vehicle control for 30 min, stimulated with SPC for 15 min, fixed, permeabilized, and stained with anti-Rho-kinase 2 antibody (green) and Hoechst nuclear stain (blue). Arrow (a → b) indicates that the change of Rho-kinase intensity from the membrane to cytosol. Scale bar = 20 μm. (**b**) Statistical analysis of changes in the ratio of Rho-kinase membrane (M) to cytosol (C) signaling M/C denotes the ratio of the intensity in membrane to cytosol signaling. Data are expressed as the mean±SE. n ≧ 10. *P < 0.01 versus vehicle control group; ^#^*P* < 0.01 versus SPC-treated group.

We previously demonstrated that translocation of Rho-kinase from the cytosol to the cell membrane plays an important role in SPC-induced Ca^2+^-sensitization of VSM contraction^8^. However, whether n-3 and n-6 DPA prevent SPC-induced contraction by suppressing Rho-kinase translocation remains unclear. Consistent with our previous report, SPC induced the translocation of Rho-kinase from cytosol to the periphery of the cell as measured by immunofluorescent staining. In contrast, Rho-kinase translocation was not observed in CASMCs pretreated with n-3 or n-6 DPA (Fig. 6a,b), suggesting that n-3 and n-6 DPA inhibited the SPC-induced translocation of Rho-kinase from cytosol to the cell membrane.

### n-3 and n-6 DPA inhibited Rho-kinase activity induced by SPC in VSM tissues and cells

**Figure 7:**
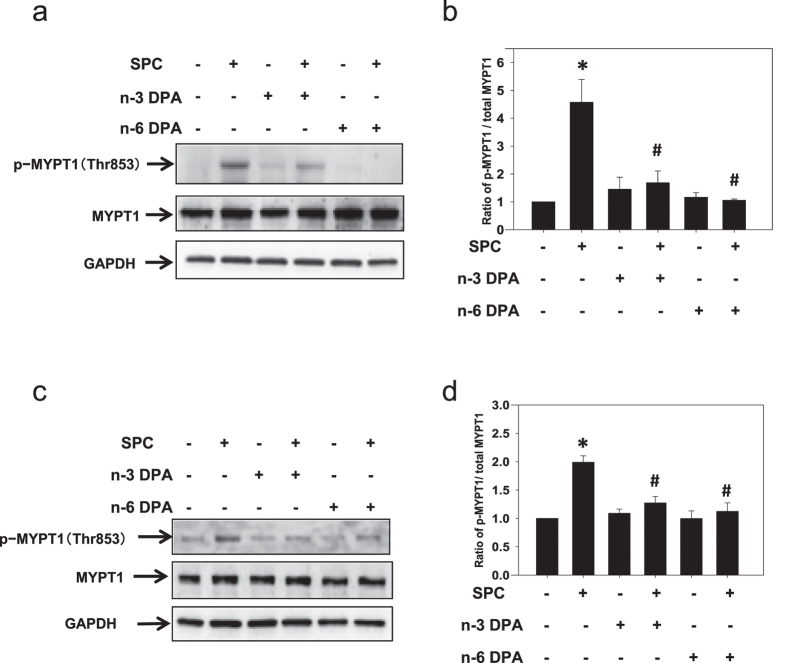
The inhibitory effects of n-3 and n-6 DPA on SPC-induced Rho-kinase activation. (**a,c**) Representative western blots using anti-p-MYPT1 (Thr853), anti-MYPT1, and anti-GAPDH antibodies from VSM tissue samples (**a**) and cells (**c**). (**b**,**d**) Statistical evaluation of the effects of n-3 and n-6 DPA (60 μM, 30 min pretreatment) on SPC-induced Rho-kinase activation, expressed as a proportion of MYPT1 phosphorylation on Thr853 to total MYPT1, and designated as 100% in the vehicle control group. *P < 0.01 versus vehicle control group; ^#^*P* < 0.01 versus SPC-treated group (n = 4).

Although n-3 and n-6 DPA inhibited SPC-induced Rho-kinase translocation, it was yet unclear whether they inhibited the SPC-induced activation of Rho-kinase. To examine if n-3 and n-6 DPA inhibited VSM contraction by preventing SPC from activating Rho-kinase, we measured threonine phosphorylation on site 853 of MYPT1 (MYPT1 Thr853) as a readout of Rho-kinase activity^34^,^35^. After stimulation with SPC, Rho-kinase activity in VSM tissues and cells increased 4.57 ± 0.82 and 1.99 ± 0.11 fold, respectively, compared with that in controls (Fig. 7). In contrast, n-3 DPA and n-6 DPA suppressed SPC-induced phosphorylation of MYPT1 Thr853 to 1.69 ± 0.42 and 1.06 ± 0.05, respectively (Fig. 7b), consistent with n-3 and n-6 DPA inhibition of SPC-induced activation of Rho-kinase. Similarly, MYPT1 Thr853 phosphorylation in VSM cells pretreated with n-3 and n-6 DPA decreased to 1.27 ± 0.11 and 1.12 ± 0.15, respectively (Fig. 7c,d).

### n-3 and n-6 DPA inhibited MLC phosphorylation induced by SPC in VSM tissue and cells

**Figure 8:**
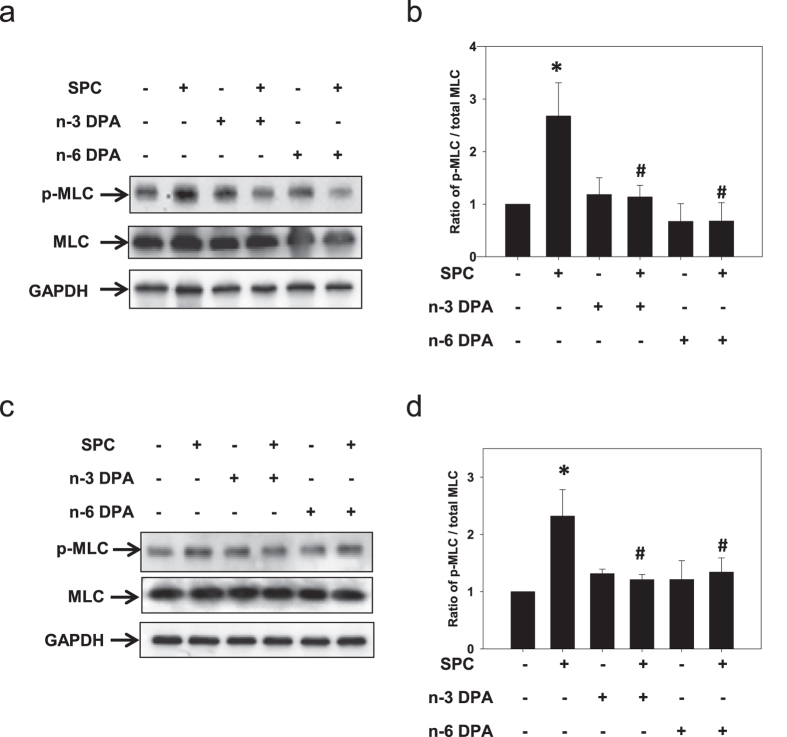
The inhibitory effects of n-3 and n-6 DPA on SPC-induced phosphorylation of MLC. (**a**,**c**) Representative western blots using anti-phospho-myosin light chain (anti-p-MLC) and anti-MLC antibodies with VSM tissue samples (**a**) and cells (**c**). (**b**,**d**) Statistical evaluation of the effects of n-3 and n-6 DPA (60 μM, 30 min pretreatment) on the level of p-MLC, which was expressed as a proportion of p-MLC to total MLC and designated as 100% in vehicle control group. *P < 0.01 versus vehicle control group; ^#^*P* < 0.01 versus SPC-treated group (n = 4).

It is known that a contraction of smooth muscle is produced when the 20-kDa myosin light chain (MLC) is phosphorylated at Ser19^36^,^37^,^38^. Activated Rho-kinase can also phosphorylate MLC at Ser19 to increase the activity of myosin ATPase *in vitro*^39^. Conversely, phosphorylation of MYPT1 by active Rho-kinase inhibits the activity of myosin light chain phosphatase (MLCP), resulting in an increase in phosphorylated MLC^40^,^41^. Because n-3 and n-6 DPA inhibited SPC-induced contraction of VSM tissue and cells, we expected that they would inhibit MLC phosphorylation. Indeed, SPC-induced phosphorylation of MLC in smooth muscle tissue pretreated with n-3 or n-6 DPA was significantly lower (1.14 ± 0.22 and 0.68 ± 0.35, respectively) compared with that in vehicle control-treated tissue (2.68 ± 0.63) (Fig. 8a,b). Similarly, both n-3 and n-6 DPA significantly inhibited phosphorylation of MLC in smooth muscle cells (Fig. 8c,d).

## Discussion

n-3 PUFAs are thought to have beneficial effects on prevention of cardiovascular diseases, while n-6 PUFAs are considered proinflammatory and thought to negatively affect the cardiovasculature. In this study, we found that both n-3 and n-6 DPA inhibited SPC-induced Ca^2+^-sensitization of VSM contraction by inhibiting the activity and translocation of Rho-kinase. To the best of our knowledge, this study is the first to show that DPA could specifically inhibit SPC-induced contraction without changing Ca^2+^ concentration. Here, we suggest that including n-6 PUFAs in the diet may be beneficial for the prevention of cardiovascular diseases arising from Ca^2+^-sensitization-mediated contraction of VSM such as in coronary artery and cerebral vasospasm. However, the reported proinflammatory effects of n-6 PUFAs need to be further investigated.

Abnormal contraction induced by SPC mediated Ca^2+^-sensitization of VSM, has been proposed as a major cause of cardio and cerebrovascular diseases such as coronary artery and cerebral vasospasm^42^,^43^. In this study, n-3 and n-6 DPA showed a much stronger inhibition of SPC-induced contraction than did the 40 mM K^+^ depolarization-induced contraction of VSM, suggesting that n-3 and n-6 DPA effectively inhibited SPC-induced abnormal contraction of VSM with little effect on Ca^2+^-dependent contraction. Furthermore, simultaneous measurement of force and [Ca^2+^]i showed that inhibitory effects of n-3 and n-6 DPA on SPC-induced contraction were Ca^2+^-independent, indicating that n-3 and n-6 DPA specifically inhibited the SPC-induced abnormal contraction of VSM. n-3 and n-6 DPA slightly inhibited the 40 mM K^+^ depolarization-induced contraction of VSM and [Ca^2+^]i, indicating that their inhibition of [Ca^2+^]i occurs by an unknown mechanism that is involved in inhibition of 40 mM K^+^ depolarization-induced contraction of VSM. Previously we showed that EPA, an n-3 PUFA, inhibited SPC-induced Ca^2+^-sensitization^8^,^10^. Thus, it is not surprising that n-3 DPA effectively inhibited the SPC-induced Ca^2+^-sensitization of VSM contraction because n-3 DPA is structurally similar to n-3 EPA. Surprisingly, the n-6 DPA, an omega-6 form of DPA, inhibited the SPC-induced abnormal contraction of VSM, providing direct evidence that n-6 PUFAs are possibly beneficial for the prevention of cardiovascular diseases.

Next, we examined the mechanisms by which n-3 and n-6 DPA inhibit SPC-induced contraction. We previously demonstrated that activation and translocation of Rho-kinase to the cell membrane are involved in SPC-induced contraction^7^. In this study, our results showed that the SPC-induced translocation of Rho-kinase to the cell membrane and its subsequent activation were significantly inhibited in cells pretreated with n-3 or n-6 DPA. Although n-3 and n-6 DPA are not shown to directly inhibit Rho-kinase activity in an *in vitro* assay, they suppressed SPC-induced Rho-kinase translocation and MYPT1 phosphorylation on the site of Thr853. Additionally, our previous study demonstrated that the SPC-induced activation of Rho-kinase is inhibited by the specific Rho-kinase inhibitor of Y27632^44^. These results suggest that SPC-induced Rho-kinase activation is inhibited by DPA through indirect suppression of Rho-kinase activity. Furthermore, SPC-induced MLC phosphorylation was significantly inhibited in both smooth muscle tissue and CASMCs pretreated with n-3 or n-6 DPA. We concluded that n-3 and n-6 DPA inhibit SPC-induced Ca^2+^-sensitization of VSM by inhibiting the activity and translocation of Rho-kinase.

Previously, we demonstrated that EPA inhibited the translocation of Fyn, a member of the Src family of tyrosine kinases (SrcPTK), which is involved in SPC-induced Ca^2+^-sensitization of VSM contraction^27^. Hence, we examined whether DPA could inhibit the translocation of Fyn, and found that DPA could not inhibit its translocation (see Supplementary Fig. S4). Although the structures of DPA and EPA are similar, the mechanisms by which they inhibit SPC-induced Ca^2+^-sensitization of VSM contraction are different. EPA is a carboxylic acid with a 20-carbon chain, while DPA has a 22-carbon chain (see Supplementary Fig. S1). The locations of the double bonds in these two compounds are also different. Further studies are needed to clarify the molecular basis of this difference.

n-3 PUFAs are metabolized *in vivo* by desaturation and elongation enzymes to form DPA and DHA, which are the major final products of EPA. n-3 DPA is formed by chain elongation of EPA performed by elongase-2 and -5, and DPA is converted to DHA by the activity of desaturase and β-oxidation^45^. n-6 DPA is the final product in the n-6 PUFA metabolism pathway. Morisaki *et al*. reported that smooth muscle cells have strong elongation activity and little or no desaturase activity^46^, suggesting that DPA may not be converted into DHA in smooth muscle. Another two studies also provide evidence that supplemented n-3 DPA shows no increase in DHA in endothelial cells^47^ and in primary hepatocytes^48^ within a period of 24 h. Additionally, Dyerberg *et al*. reported that administering linoleic acid (LLA) orally to humans for 7 days was not long enough to cause significant increases in the plasma levels of EPA and DHA^49^, suggesting that the metabolism of PUFAs *in vivo* is a very slow process. Therefore, we speculate that in the present study, there is little or no possibility of converting DPA to other vasoactive metabolites in smooth muscle tissue or cells, especially over a short period (treatment with DPA for 30 min).

**Figure 9:**
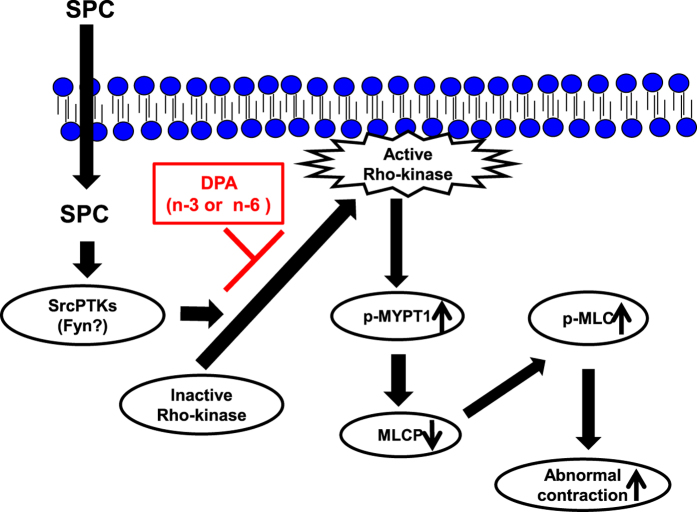
Proposed mechanism by which n-3 and n-6 DPA inhibits SPC-induced Ca^2+^-sensitization of VSM contraction by inhibiting activation and translocation of Rho-kinase. SPC stimulation causes SPC-induced Ca^2+^-sensitization and abnormal contraction of VSM via the SPC/SrcPTKs/Rho-kinase pathway. Additionally, SPC-induced translocation of Rho-kinase from cytosol to the cell membrane plays a vital role in SPC-induced contraction. Here, we demonstrated that n-3 and n-6 DPA inhibit SPC-induced Ca^2+^-sensitization of VSM contraction via inhibiting the activation and translocation of Rho-kinase.

In conclusion, the results of our present study demonstrated that two forms of DPA (n-3 and n-6) inhibit SPC-induced Ca^2+^-sensitization of VSM contraction. This inhibition occurs via suppression of SPC-induced Rho-kinase activation and translocation to the cell membrane, and subsequent inhibition of MLC phosphorylation (Fig. 9). Importantly, we also found that n-6 DPA effectively inhibits SPC-induced Ca^2+^-sensitization of VSM contraction. This study may provide new insights for the prevention of cardiovascular and cerebrovascular vasospasm by n-6 PUFAs.

## Materials and Methods

### Reagents and antibodies

n-3 DPA (purity ≧ 98%) solution in 100% ethanol was obtained from Cayman Chemical (Ann Arbor, MI, USA). n-6 DPA (purity > 99%) was purchased from Nu-Chek Prep, Inc. (Elysian, MN, USA). DPA was dissolved in 100% ethanol to make a 30 mM stock solution. The stock solutions were stored at −20 °C, then diluted to final concentrations before use. Sphingosylphosphorylcholine (SPC) was purchased from Biomol (Plymouth Meeting, PA, USA). Fura-2/AM was purchased from Dojindo (Kumamoto, Japan). Bradykinin (BK) was purchased from Peptide institute, Inc. (Osaka, Japan). All other chemicals were purchased from Katayama Chemical (Osaka, Japan).

The following antibodies were used: anti-phospho-MYPT1 (Thr850) with phosphorylation site corresponding to Thr853 in human MYPT1 (Upstate), anti-GAPDH monoclonal antibody (Wako), anti-phospho-myosin light chain 2 (Ser19) monoclonal antibody (Cell Signaling), anti-myosin light chain (20 kDa) monoclonal antibody (Sigma Aldrich), anti-Rho-kinase-2 polyclonal antibody (H85, Santa Cruz), and anti-rabbit Alexa Fluor 488 (Thermo Fisher). Secondary HRP-labeled antibodies (anti-mouse and anti-rabbit) were purchased from Promega.

### Cell culture

Human coronary artery smooth muscle cells (CASMCs) were cultured in HuMedia SG2 (Kurabo, Japan) supplemented with 5% fetal bovine serum (FBS), 0.5 ng/ml human epidermal growth factor (hEGF), 2 ng/ml human fibroblast growth factor-B (hFGF-B), 5 μg/ml insulin, 50 μg/ml gentamycin, and 50 ng/ml amphotericin B. Cells were maintained at 37 °C in a humidified atmosphere of 5% CO_2_ and 95% air. Human CASMCs were used for experiments at passage numbers <10 splitting cycles.

### Preparation of VSM strips

All the procedures were subject to approval by the Institutional Animal Care and Use Committee of Yamaguchi University and were conducted in conformity with institutional guidelines. Porcine left anterior descending arteries were obtained from a public abattoir (Kitakyushu Municipal Meat Inspection and Control Center, Japan). Tissue specimensof coronary arteries were placed into ice-cold Krebs solution (123 mM NaCl, 4.7 mM KCl, 15.5 mM NaHCO_3_, 1.2 mM KH_2_PO_4_, 1.2 mM MgCl_2_, 1.25 mM CaCl_2_, and 11.5 mM D-glucose) for transport to our laboratory. An optical microscope was used to view fat tissue and adventitia, which were removed with minimum stretch to VSM. The arteries were cut into helical strips lacking endothelium. The complete removal of the endothelium from strips was confirmed by the lack of any relaxation response to 1 μM BK.

### Measurement of force in VSM

Measurements of isometric force in VSM strips (1.0 × 4.0 mm) were carried out as described previously^32^,^33^. Briefly, after recording steady responses to repeated applications of 118 mM K^+^-depolarization, an injection of 30 μM SPC or 40 mM K^+^ was used to induce a contraction of VSM. Then, the effects of n-3 or n-6 DPA were examined at the plateau phase of the sustained contraction induced by 30 μM SPC or 40 mM K^+^. The inhibitory extent of contraction induced by n-3 or n-6 DPA was described as a percentage of the response to the contraction induced by 30 μM SPC or 40 mM K^+^ (see Supplementary Fig. S2).

### Simultaneous Measurement of [Ca^2+^]i and contraction of VSM

We measured the changes in [Ca^2+^]i and force simultaneously using porcine coronary arterial strips as described previously^7^. Briefly, strips of porcine coronary arteries were loaded with 12.5 μM Fura-2/AM for 4 h at 37 °C. Then, changes in [Ca^2+^]i were continuously monitored using a spectrofluorometer (CAM230, JASCO) to assess fluorescence intensity at excitation of 340 nm (F340) and 380 nm (F380) as well as the fluorescence ratio (r = F340/F380).

### Immunofluorescent staining

For immunofluorescence studies, human CASMCs were cultured on 0.3% gelatin-coated glass coverslips. After stimulation with SPC, cells were fixed in 2% paraformaldehyde in phosphate buffered saline (PBS) for 10 min, permeabilized with 0.1% Triton X-100 in PBS for 2 min, and blocked in NanoBio blocker solution (Nano Bio Tech Co., Ltd) diluted in PBS for 60 min at room temperature. Cells were then incubated with the primary anti-Rho-kinase-2 antibody for 60 min. The antigen-antibody complex was visualized with Alexa Fluor 488-conjugated secondary antibody (Thermo Fisher Scientific). Coverslips were washed with PBS, rinsed with deionized water, and mounted with PermaFluor aqueous mounting medium (Thermo Fisher Scientific). Specimens were observed using fluorescent microscopy (KEYENCE, Japan).

### Western blotting analysis

Tissues were preincubated with n-3 or n-6 DPA (60 μM) in Krebs solution at 37 °C. After 30 minutes, tissues were stimulated with SPC (30 μM for 15 min at 37 °C) in the absence (vehicle control) or presence of DPA in Krebs solution. Then, tissues were rapidly frozen in 10% trichloroacetic acid (TCA)/10 mM DL-dithiothreitol (DTT) in acetone pre-chilled on ice and washed twice with cold 10 mM DTT/acetone. Tissues were then placed into liquid nitrogen and crushed using the SK-Mill Freeze-Crush Apparatus (Funakoshi, Japan). Protein was extracted from freeze-dried tissues by the addition of 100 μl RIPA buffer (Wako) and protease inhibitor cocktail (Sigma), and samples were kept at 95 °C for 5 min for western blot analysis.

Cell lysates were prepared as follows. Human CASMCs were serum starved for 24 h, then stimulated with SPC (30 μM for 15 min) with pretreatment with n-3 or n-6 DPA (60 μM for 30 min) or vehicle control. Cells were lysed in lysis buffer (50 mM Tris-HCl, pH 7.4; 150 mM NaCl; 0.5% NP-40; 0.1% Triton X-100; 1 mM DTT; 0.2 mM Na_3_VO_4_; 10 mM NaF; 1 mg/ml aprotinin; and protease inhibitor cocktail (Sigma)). The lysates were centrifuged, and the supernatants were boiled in sodium dodecyl sulfate (SDS) loading buffer. Tissue or cell samples were separated using 10% SDS-polyacrylamide gel electrophoresis, transferred to Amersham Hybond-P PVDF membrane (GE Healthcare Life Sciences), and blocked in 5% non-fat milk in 0.05% Tween-20 phosphate-buffered saline (TBS-T) for 60 min at room temperature. The membranes were incubated with primary antibodies for 1 h at room temperature or overnight at 4 °C, followed by incubation with peroxidase-conjugated secondary antibodies for 1 h at room temperature. Immunoreactive bands were detected using a Supersignal West Pico Chemiluminescent Substrate kit (Thermo Fisher Scientific). Glyceraldehyde 3 phosphate dehydrogenase (GAPDH) was used as loading control.

### Time-lapse recording of VSM cell contraction

Human CASMCs were grown in 35-mm glass-based dishes (Iwaki, Japan). When cell confluence reached 90–100%, FBS and growth factor-free HuMedia SB2 (Kurabo, Japan) medium was changed to obtain the hypercontractile type of CASMCs according to previously existing methods^34^,^35^. After treatment with HuMedia SB2 for 48 h, cells were pretreated with n-3 or n-6 DPA (60 μM) for 30 min at 37 °C. Then, 30 μM SPC was added to the medium and time-lapse recording of VSM cell contraction was performed under the fluorescent microscope (KEYENCE, Japan).

### Automated image capture and analysis

Human CASMCs were seeded in 96-well plates. Before stimulation with SPC, media were removed and cells were stained with 5-chloromethylfluorescein diacetate (Cell-Tracker Green CMFDA; Molecular Probe) at a final concentration of 1 μM in serum-free HuMedia SB2 (Kurabo, Japan). After 30 min incubation at 37 °C, the dye was removed, replaced with fresh, pre-warmed HuMedia SB2, and incubated for 30 min at 37 °C. After pretreatment with DPA (60 μM for 30 min at 37 °C), cells were stimulated with SPC. Then, cells were fixed in 7.4% formaldehyde (Sigma Aldrich) in PBS containing Hoechst 33258 nuclear stain (Thermo Fisher Scientific) for 15 min at room temperature. The fixation buffer was removed and replaced with 100 μl PBS. Plate analysis and image acquisition were performed immediately using ArrayScan V^TI^ High Content Screening Reader (Thermo Fisher Scientific, USA). Images were acquired in two channels: channel 1 for the cytoplasmic chloromethyl fluorescein diacetate (CMFDA) stain and channel 2 for the Hoechst nuclear stain. The parameters set for nuclei recognition ensured that any artifacts or cell clusters were absent in the populations analyzed. The images were automatically stored and analyzed by the ArrayScan™ Software; at least five fields were analyzed in each well with a 10X objective, corresponding to at least 1,000 cells counted.

### Statistical Analysis

All data are expressed as mean ± SEM. Differences between groups were analyzed by Student’s t-test. Values of *P* < 0.05 were considered to represent a statistically significant difference.

## Additional Information

**How to cite this article**: Zhang, Y. *et al*. Omega-3 and omega-6 DPA equally inhibit the sphingosylphosphorylcholine-induced Ca^2+^-sensitization of vascular smooth muscle contraction via inhibiting Rho-kinase activation and translocation. *Sci. Rep.* **7**, 36368; doi: 10.1038/srep36368 (2017).

**Publisher's note:** Springer Nature remains neutral with regard to jurisdictional claims in published maps and institutional affiliations.

## Supplementary Material

Supplementary Information

Supplementary Video 1

Supplementary Video 2

Supplementary Video 3
