# Supplementary material for: Omega-3 and omega-6 DPA equally inhibit the sphingosylphosphorylcholine-induced Ca2+-sensitization of vascular smooth muscle contraction via inhibiting Rho-kinase activation and translocation
Source: Sci Rep. 2017 Feb 7;7:36368. doi: 10.1038/srep36368 (PMC5294466; doi:10.1038/srep36368)
Supplement: Supplementary Information [file srep36368-s1.pdf]

Omega-3 and omega-6 DPA inhibits Ca^{2+}-sensitization of vascular smooth muscle contraction induced by sphingosylphosphorylcholine via inhibiting Rho-kinase activation and translocation

Ying Zhang, Min Zhang, Bochao Lyu, Hiroko Kishi, and Sei Kobayashi*

Department of Molecular and Cellular Physiology, Yamaguchi University, Graduate School of Medicine, 1-1-1 Minami-Kogushi, Ube, Yamaguchi 755-8505, Japan

* Address all correspondence and requests for reprints to: Sei Kobayashi， Department of Molecular and Cellular Physiology, Yamaguchi University, Graduate School of Medicine, 1-1-1 Minami-Kogushi, Ube, Yamaguchi 755-8505, Japan

Tel: (81)-836-22-2209; Fax: (81)-836-22-2348

E-mail address: seikoba@yamaguchi-u.ac.jp

Supplementary Video S1 Time-lapse video of SPC-induced contraction of vascular smooth muscle cells. Images were acquired at 30 second intervals for 30 min.

Supplementary Video S2 Time-lapse video of SPC-induced contraction of vascular smooth muscle cells pretreated with n-3 DPA (60 μM) for 30 min. Images were acquired at 30 second intervals for 30 min.

Supplementary Video S3 Time-lapse video of SPC-induced contraction of vascular smooth muscle cells pretreated with n-6 DPA (60 μM) for 30 min. Images were acquired at 30 second intervals for 30 min.

Supplementary Fig.S1

The chemical structures of n-3 DPA , n-6 DPA and EPA.

n-3 DPA

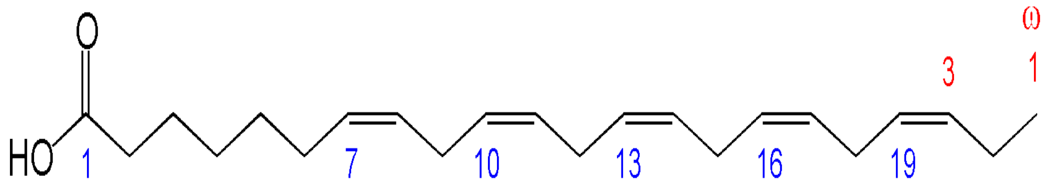

n-6 DPA

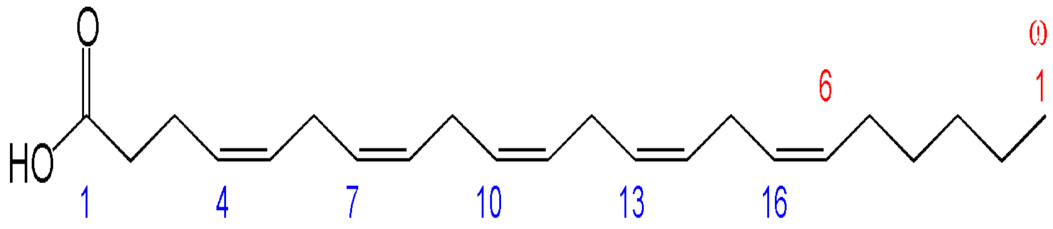

EPA

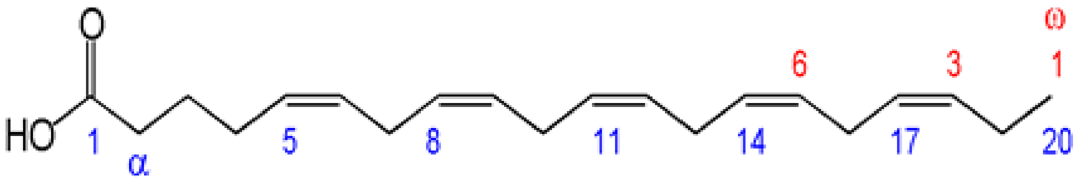

Supplementary Fig.S2

a SPC-induced contraction in the presence of vehicle.

b 40 mM K^+-induced contraction in the presence of vehicle.

c The inhibitory extent of contraction induced by n-3 DPA or n-6 DPA was calculated by a percentage of the response to the contraction induced by 30 μM SPC.

a

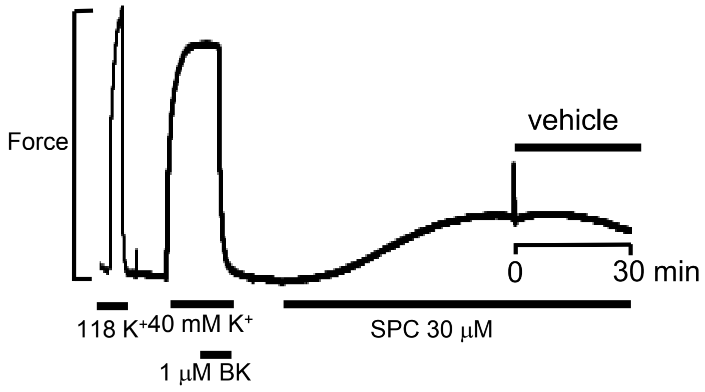

b

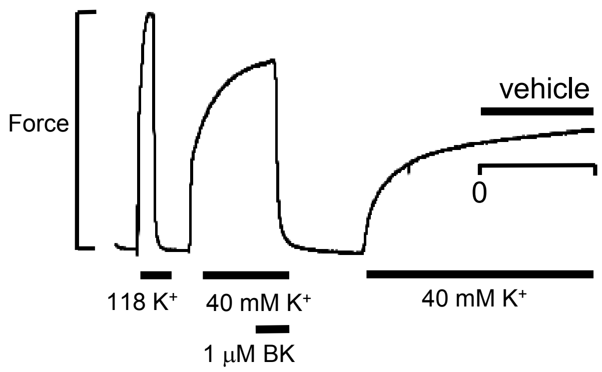

c

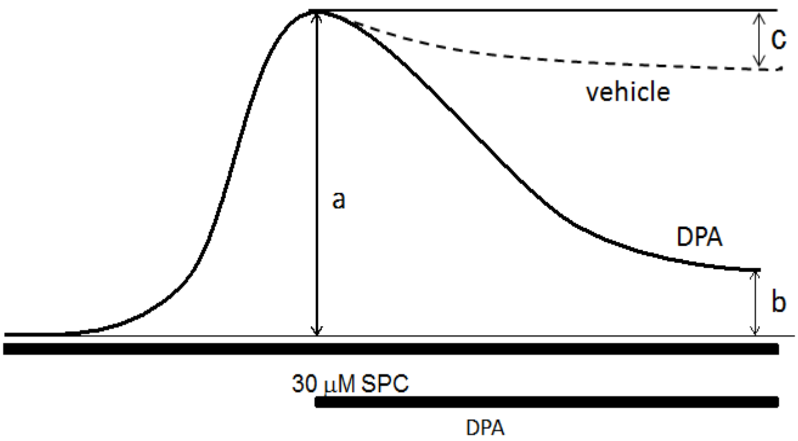

a: The contraction of VSM induced by SPC.
b: The contraction of VSM in the presence of DPA.
c: The contraction of VSM in the presence of vehicle.
Inhibitory ratio of DPA (%) = (a-b-c)/a × 100%.

Supplementary Fig.S3

Induction of contractile type of human CASMCs after serum-free medium treatment for 2 days. Scale bar =200 μm.

a Phase-contrast micrograph of human CASMCs in normal medium.

b Phase-contrast micrograph of human CASMCs in serum-free medium for 2 days.

a

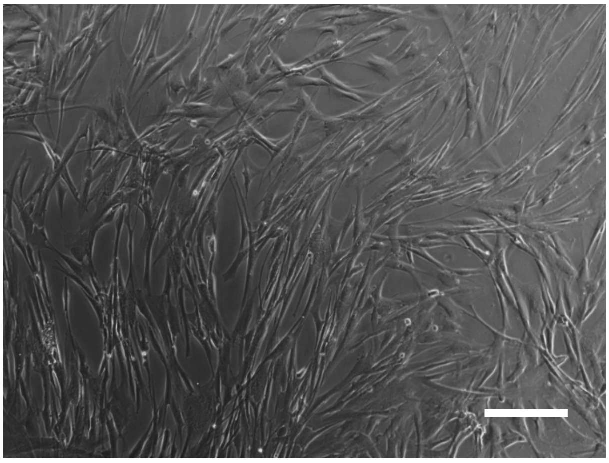

b

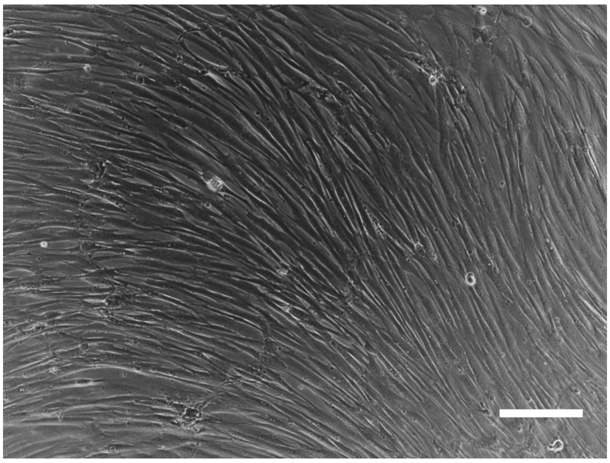

Supplementary Fig.S4

a Control image of immunofluorescent staining in the presence of second antibody. Scale bar = 10 μm.

b Translocation of Fyn induced by SPC in the presence of EPA and DPA. Scale bar = 100 μm.

a

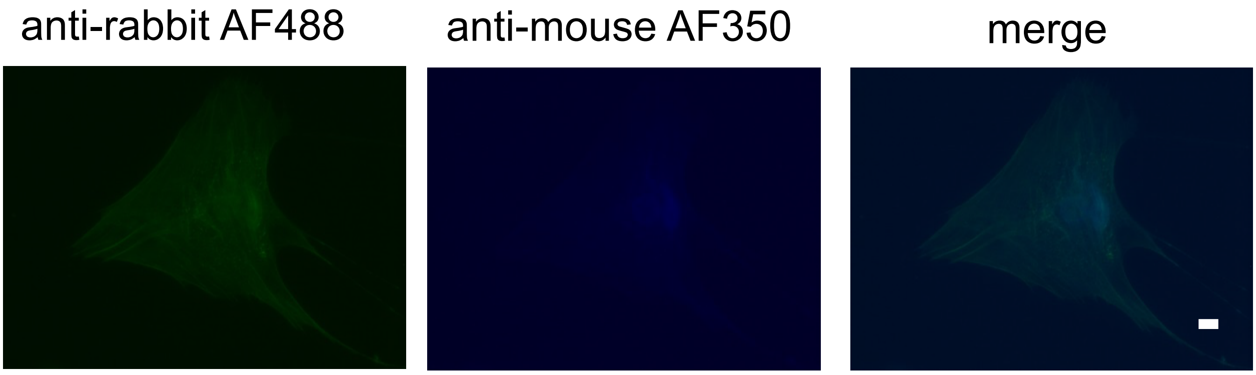

b

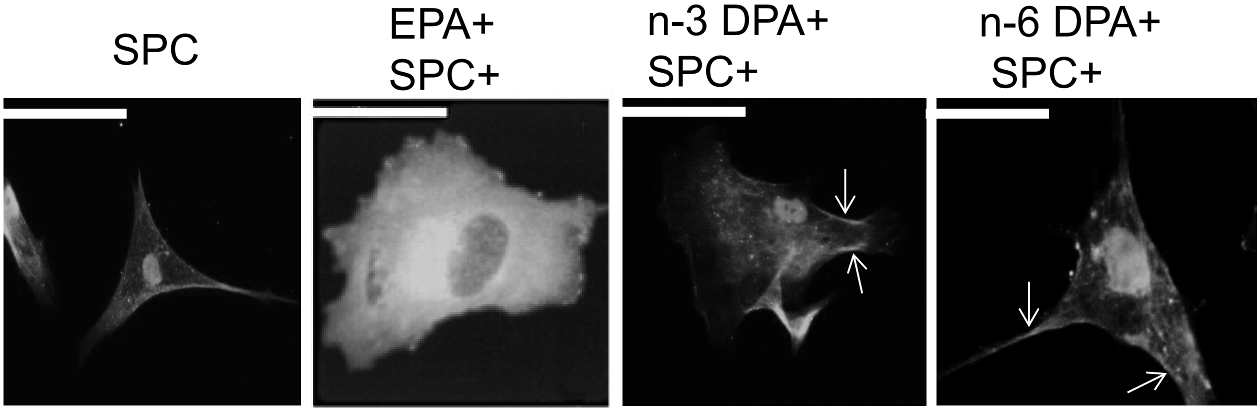

As a reference, the picture (EPA+ SPC+) obtained from our previous publication (Ref. 27) is shown.
